# Procedural performance following sleep deprivation remains impaired despite extended practice and an afternoon nap

**DOI:** 10.1038/srep36001

**Published:** 2016-10-26

**Authors:** Irma Triasih Kurniawan, James Nicholas Cousins, Pearlynne L. H. Chong, Michael W. L. Chee

**Affiliations:** 1 Centre for Cognitive Neuroscience, Duke-NUS Medical School, Singapore, 169857, Singapore; 2 Motivation, Brain, & Behavior (MBB) team, Institut du Cerveau et de la Moelle Epinière (ICM), Université Pierre et Marie Curie (UPMC – Paris 6), 75013 Paris, France

## Abstract

The negative impact of sleep loss on procedural memory is well established, yet it remains unclear how extended practice opportunities or daytime naps can modulate the effect of a night of sleep deprivation. Here, participants underwent three training and test conditions on a sequential finger tapping task (SFTT) separated by at least one week. In the first condition they were trained in the evening followed by a night of sleep. Two further conditions took place where evening training was followed by a night of total sleep deprivation (TSD). One of the TSD conditions included a one-hour nap opportunity (15:00). Compared to the condition in which sleep was permitted, a night of TSD resulted in poorer performance across 4 practices the following day (10:00–19:00). The deleterious effect of a single night of TSD on procedural performance, was neither clearly alleviated by an afternoon nap nor by multiple practice opportunities. Interestingly, significant gains in performance were observed in all conditions after a one-week delay. Recovery sleep on subsequent nights thus appeared to nullify the effect of a single night of sleep deprivation, underscoring the importance of offline consolidation on the acquisition of procedural skill.

Sleep is widely regarded as critical to the efficient function of a number of cognitive operations^1^, particularly learning and memory^2^. Given that a large number of adults are obtaining inadequate sleep^3^, there is a growing need to accurately characterise the detrimental effects of sleep loss on learning and to identify effective countermeasures.

The acquisition and consolidation of procedural skills has been studied extensively using the sequential finger tapping task (SFTT), an explicit motor sequence learning (MSL) task that measures the speed and accuracy of repeatedly performing a short sequence of finger movements^4^,^5^. This type of learning progresses primarily via repeated practice^6^, but delayed offline gains also occur following nocturnal sleep^7^,^8^ or a daytime nap^9^,^10^,^11^,^12^.

The aforesaid MSL studies typically incorporate a training period prior to a sleep or wake retention interval, followed by testing to gauge sleep-dependent gains in performance^8^. These post-retention tests are typically very short^8^,^10^,^11^,^13^,^14^,^15^, to facilitate the assessment of consolidation in a manner that minimizes the possible contribution of repeated practice, fatigue or reaching a ceiling in performance. Thus, it remains unclear the extent to which multiple practice opportunities might reduce the negative impact of total sleep deprivation (TSD) on learning.

Further, the long-term consequences of sleep deprivation on procedural memory beyond the initial 24–48 hours of learning^9^,^10^,^12^,^16^ have not been explored. Of interest, a recent study suggested that a week of recovery sleep after a night of sleep deprivation may facilitate recovery from impairment in MSL performance through delayed memory consolidation^17^.

Here we used a MSL task to test the effect of TSD on procedural performance over extended practice opportunities spanning a day. Participants were trained on the SFTT in the evening prior to a night of sleep or TSD. The following day they performed four additional practices at 10:30, 13:30, 17:30 and 19:00, with a final test after one week. We predicted that sleep would provide superior offline performance gains relative to TSD, and that performance would continue to lag in the TSD conditions across practices throughout the day. Next, to assess whether a daytime nap could alleviate this impairment, participants performed two TSD conditions one-week apart that either incorporated a one-hour daytime nap (15:00) or did not. We predicted that a nap would attenuate the negative effects of a night of TSD on motor sequence performance. Finally, we predicted a persistent effect of TSD on procedural memory after one week.

## Results

**Figure 1:**
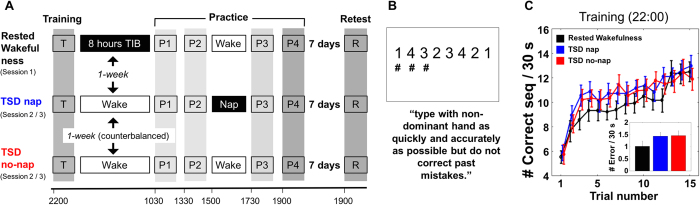
(**A**) Schematic showing layout of experimental conditions involving training (T), next-day practice (P1-P4), and one-week retest (R). In Rested Wakefulness condition, participants completed training at home and had 8 hours time-in-bed (TIB) before entering the laboratory the following day at 10:00. The following two conditions were TSD conditions where participants arrived at the laboratory at 18:00, were trained on new sequences, and remained awake overnight. A one-hour nap opportunity was provided at 15:00 for one of the TSD conditions (order counterbalanced). Training and practices involved 15 trials of 30-sec sequence performance followed by 30-sec rest periods. Practice 4 was followed by 3 trials of a novel sequence with rest periods. The one-week retest consisted of 3 trials with rest periods. For each participant, three different learned sequences were randomly assigned to the three conditions, as were three novel sequences. (**B**) MSL task display and instructions. (**C**) Performance speed in training across trials 1–15. Inset. Number of incorrect sequences made within 30-sec. Mean ± SEM, there were significant differences between RW versus both TSD conditions, indicating a sequence-independent practice effect after the first experimental condition.

Participants underwent three experimental conditions separated by at least one week, during which they learned a new sequence (Fig. 1A). In the ‘rested wakefulness’ (RW) condition, which was always the first condition undertaken, training was followed by a night of sleep. This control condition was always performed first. As such, sequence independent learning that could benefit performance speed was expected to take place primarily in this first session, allowing comparison of subsequent TSD nap and no-nap conditions with limited influence of such sequence independent learning. In the second and third conditions, participants underwent a night of TSD supervised by a research assistant. One of these TSD conditions included a one-hour nap opportunity at 15:00. The order of TSD conditions was counterbalanced. The number of correct sequences completed (speed) and the number of incorrect responses made (error) within a 30-sec trial were measured.

### Training

Participants successfully learned a new sequence during the training phase of all experimental conditions. A two-way repeated measures ANOVA with condition (RW/TSD nap/TSD no-nap) and trial (1–15) showed a main effect of trial (*F*(14,238) = 46.73, *p* < 0.0001; Fig. 1C), and performance was significantly faster for the mean of the last three training trials relative to the first three trials (*t*(17) = 15.88, *p* < 0.0001).

There was a condition-related difference in training performance between the initial RW to the latter two TSD conditions (Fig. 1C). Participants were slower (*t*(17) = 2.89, *p* = 0.01) but more accurate (*t*(17) = 2.36, *p* = 0.03) in the RW condition, compared to the TSD conditions (main effect of condition: *F*(2,34) = 4.54, *p* = 0.017; condition-by-trial interaction: *F*(28,476) = 1.36, *p* = 0.1). This order-related improvement plateaued after the first condition (RW), and there were no further gains between the two TSD conditions (*t*(17) = 0.6, *p* = 0.5). Comparing the training performance across the first and second TSD visits regardless of nap assignment also yielded a non-significant difference (*t*(17) = 0.06, *p* = 0.9). Importantly, training performance in the last three trials were comparable across conditions (one-way repeated measures ANOVA, main effect of condition: *F*(2,34) = 0.5, *p* = 0.61), confirming that performance had reached the same level prior to the sleep manipulation.

### TSD effect on procedural performance

Changes in performance across day 2 were calculated as a percentage relative to the average speed during the last 3 trials of training. First, to explore overnight consolidation effects, we examined the change in performance on the first three trials in Practice 1. Conditions were compared via a one-way repeated measures ANOVA, revealing a comparable drop in performance across conditions (main effect of condition: *F*(2,34) = 0.1, *p* = 0.89; *M*_RW_ = −3.6 ± 3%; *M*_TSD_nap_ = −5.4 ± 2.5%, *M*_TSD_no-nap_ = −5.3 ± 3.6%; paired-samples t-tests on RW vs TSD Nap: *t*(17) = 0.39, *p* = 0.6; RW vs TSD No-nap: *t*(17) = 0.3, *p* = 0.7).

Next we assessed performance improvement across all practices. Here, the percentage change in performance was computed using the average performance across all 15 trials in each practice (P1-P4). A two-way repeated measures ANOVA with condition (RW/TSD nap/TSD no-nap) and practice (P1-P4) was then performed. Follow-up tests were performed to test the effects of condition in each practice separately. We also ran a priori set of contrasts by collapsing performance across some practices in order to explore performance trends across the day and the influence of napping. Also TSD conditions were sometimes collapsed for comparisons to RW.

**Figure 2:**
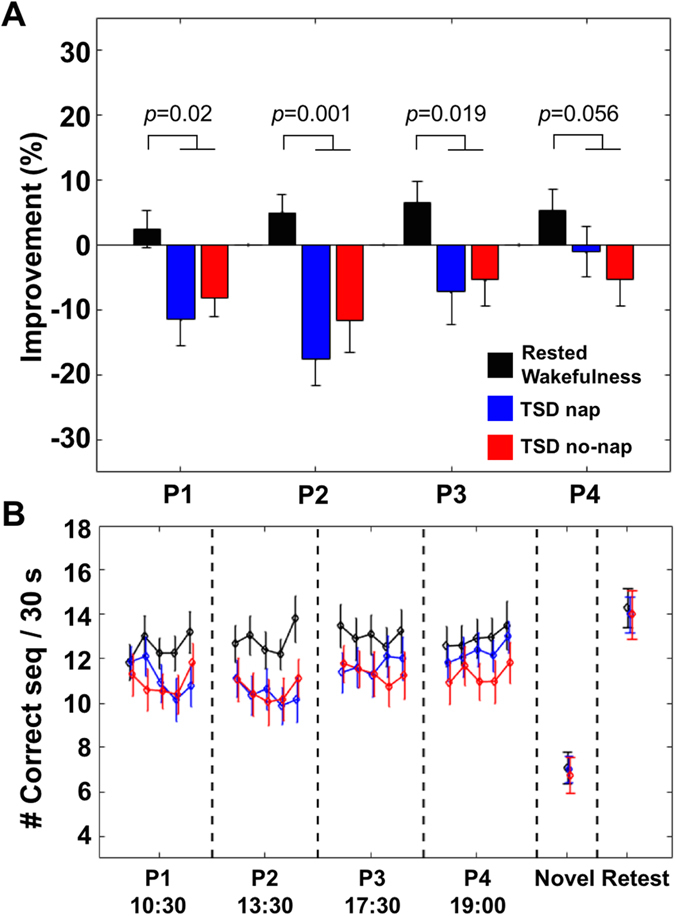
(**A**) TSD resulted in lower levels of improvement relative to the last 3 trials of training performance, across mean performance during practices at 10:30 (P1), 13:30 (P2), 17:30 (P3) and 19:00 (P4). Improvement did not differ between TSD nap and no-nap; p values are for paired-samples t-tests between RW and average of TSD conditions. (**B**) The trajectory of absolute performance levels within and across practices showed consistently worse performance for TSD conditions compared to the RW condition. Five data points within each practice represent mean performance on bins of 3-trials (trials 1–3, 4–6, 7–9, 10–12, and 13–15) and are included solely for illustrative purposes. Performance of a novel sequence at the end of Practice 4 (Novel) did not differ across conditions. There were significant overall gains in performance after one-week (Retest), but this improvement did not differ between conditions. Mean ± SEM.

Across the day, the RW condition remained at a consistently higher level than TSD conditions (Fig. 2A) (main effect of condition: *F*(2,34) = 4.39, *p* = 0.002; RW > TSD conditions, *t*(17) = 3.04, *p* = 0.0073; main effect of practice: *F*(3,51) = 7.56, *p* < 0.0001). A significant practice-by-condition interaction (*F*(6,102) = 2.95, *p* = 0.01) showed that performance differences between conditions varied across practices. In practices 1–3, follow-up tests demonstrated significantly faster RW performance compared to collapsed TSD conditions (P1: *t*(17) = 2.56, *p* = 0.02; P2: *t*(17) = 3.87, *p* = 0.0012; P3: *t*(17) = 2.57, *p* = 0.019), while this comparison was marginal for P4 (*t*(17) = 2.05, *p* = 0.056). Performance in TSD conditions did not significantly differ for any practice (*p*s > 0.34). These findings indicate a negative effect of TSD on finger tapping performance across multiple practices, with only marginal recovery in the final practice session.

To explore whether the daytime nap improved TSD performance, a set of a-priori contrasts were made between collapsed pre and post-nap practices (P1 and P2 vs. P3 and P4). Both TSD conditions showed significantly faster performance in the second half of the day (P3 and P4 > P1 and P2; Nap: t(17) = 4.05, p < 0.0001; No-nap: t(17) = 2.17 p = 0.04). In contrast, RW performance did not significantly improve in the second half of the day (t(17) = 1.74, p = 0.09). This was supported by a significantly larger size of improvement ([P3 and P4]) minus ([P1 and P2]) in the collapsed TSD conditions compared to that in the RW condition (*t*(17) = 3.36, *p* = 0.003). However, the size of such improvement did not significantly differ between the TSD conditions (*t*(17) = 1.71, *p* = 0.1). Critically, the ‘performance recovery’ across the day in the TSD condition was not significantly modulated by nap (Fig. 2A).

Importantly, we found a similar performance profile across conditions when participants performed a novel sequence for three 30-sec trials at the end of each condition (Fig. 2B (novel); main effect of condition: *p* = 0.5). Thus, the observed TSD effects appear to be specific to trained sequences, suggesting the impaired performance after TSD was unlikely to be a result of simple motor slowing.

A decline in performance within practice episodes could indicate reactive inhibition^18^, referring to performance decline as a result of boredom or fatigue. This led us to explore whether participants were more susceptible to these effects after TSD by performing additional subject wise regression analyses (Supplementary Fig. 1). These showed that TSD performance within each practice did not decline more rapidly than that in the RW condition (two-way repeated measures ANOVA, main effect of condition and condition-by-practice interaction, *p*s > 0.05), suggesting that build-up of fatigue over time during practice did not differ across conditions.

A week later, without additional practice, there was a significant improvement in performance relative to the last three trials of practice 4 in all conditions (*p*s < 0.005). The magnitude of improvement was comparable across conditions (*M*_RW_ = 11.9 ± 7.3%; *M*_TSD_nap_ = 9 ± 4.5%, *M*_TSD_no-nap_ = 22.4 ± 8.3%; one-way repeated measures ANOVA, main effect of condition: *p* = 0.4). Moreover, improvement relative to the last three trials of training also showed no significant differences across conditions (*M*_RW_ = 18.2 ± 4.6%; *M*_TSD_nap_ = 12.5 ± 4.2%, *M*_TSD_no-nap_ = 17.3 ± 4.3%; one-way repeated measures ANOVA, main effect of condition: *p* = 0.6), suggesting that the negative effects of TSD were not present after one-week of offline consolidation.

### Psychomotor Vigilance Task (PVT)

This was performed to confirm an expected decline in vigilance following TSD. As expected, a 10-min PVT administered immediately after practice 1 (10:30) at 11:00 indicated that reciprocal RT (1/RT) was significantly worse following TSD nights compared to a night of sleep (one-way repeated measures ANOVA; main effect of condition, *F*(2,32) = 26.9, *p* < 0.0001; RW > TSDs; *t*(16) = 6.02, *p* < 0.0001; Supplementary Table 3). TSD nap and no-nap conditions did not differ (*t*(16) = 0.4, *p* = 0.6). However, this impaired vigilance (RW minus averaged TSD conditions) did not significantly correlate with the impairment in SFTT performance at practice 1 (RW minus TSD) (*r*(16) = 0.23, *p* = 0.3; Supplementary Fig. 2).

A further 3-minute PVT was performed 30-minutes after the conclusion of the nap, primarily to assess any differences in sleep inertia between TSD nap and TSD no-nap conditions. Again this showed impaired vigilance (one-way repeated measures ANOVA; main effect of condition, *F*(2,34) = 8.26, *p* = 0.0012; RW > TSDs; *t*(17) = 4.58, *p* < 0.0001; Supplementary Table 3), but TSD nap and no-nap conditions did not differ (*t*(17) = 1.1, *p* = 0.2). The correlation between impaired vigilance and impaired SFTT performance during practice 3 one hour later (17:30) (RW minus TSD) was non-significant (*r*(17) = 0.4, *p* = 0.06; Supplementary Fig. 3). Together these tests suggest that the TSD effect on alertness cannot account for the persistently inferior SFTT performance.

### Nap quality

PSG data (Table 1) for the one-hour nap showed that participants fell asleep within 6.9 minutes of lights out, had a high sleep efficiency (90%) and obtained an average of 43 minutes of slow wave sleep (SWS). Out of 18 participants, 10 were awoken from SWS, 5 from stage 2 sleep, 2 from REM sleep, and 1 was already awake.

## Discussion

Consistent with previous findings^7^,^16^,^17^, we found that a single night of total sleep deprivation (TSD) impaired procedural task performance the following day. Furthermore, the effect of TSD in depressing performance persisted over multiple practice episodes throughout that day and was not clearly alleviated by a one-hour afternoon nap. Interestingly, regardless of whether participants slept normally or were sleep deprived, performance significantly improved after a week despite participants having no additional practice. Thus, subsequent offline consolidation appeared to be sufficient to nullify the effect of a single night of sleep deprivation on procedural memory.

We observed a significant impairment in MSL performance on Practice 1 following the overnight TSD manipulation. This difference in performance held across all practices that day. A detailed exploration of the underlying mechanisms of impaired performance is beyond the scope of the current study, but a likely candidate is the missed opportunity for sleep-dependent consolidation of the motor sequence. A large body of research has demonstrated sleep-dependent performance improvement during a short post-retention test^7^,^16^,^19^,^20^. Adding to this, our observations suggest that the benefits offered by sleep extend across multiple practice opportunities the day following learning.

Non-specific motor slowing could, in theory, have contributed to the observed TSD impairment. Prior work has identified reactive inhibition as an influential factor in the SFTT^18^,^13^, whereby performance progressively worsens across continuous training sessions and can be recovered with breaks. Potentially, sleep loss exacerbates the negative impact of these sequence-independent factors. However, we found no difference between conditions in the decline of performance within each round of practice, suggesting that reactive inhibition was not compounded by TSD in this experiment. This is likely a result of the relatively short bursts of testing (30 s) separated by breaks. It is known that breaks can reset time-on-task effects even in the sleep deprived state^21^. Furthermore, the performance of a novel sequence introduced after the final practice did not differ across conditions, indicating that any detriment to performance after TSD was specific to the learned sequence.

Impaired vigilance after TSD could also potentially contribute to a non-specific slowing, whereby attentional lapses reduce performance levels rather than motor slowing per se. As expected, we observed reduced vigilance for TSD conditions relative to RW for PVTs performed at 11:00, but this impairment did not correlate significantly with impaired SFTT performance. However, as there was a trend for the association between TSD effects on PVT performance and SFTT in the afternoon (16:30), it remains possible that the decrease in alertness had an influence on SFTT performance. Future studies could systematically assess the time course of effects of TSD on SFTT performance and alertness levels.

Together these findings suggest that non-specific motor slowing attributable to fatigue did not materially contribute to impaired performance of the SFTT after a night of sleep deprivation. Instead, the impairment appears to be specific to learned sequences.

Alternatively, impairment after TSD may relate to faulty post-retention learning, whereby re-encoding of the sequence representation was impaired across practices in the sleep-deprived state. However, the lack of additional learning across all practices in the RW condition suggests there was minimal post-retention learning the following day.

Interestingly, we did not find the overnight sleep ‘enhancement’ observed in previous studies when considering the first 3 trials of performance at Practice 1^7^,^16^,^8^,^10^. This null observation is in fact consistent with more recent findings, where short rest periods without sleep provide an early boost to performance, which then negates any subsequent overnight enhancement on the SFTT^13^,^14^,^15^. In light of this, sleep is considered to stabilize performance against decay across wakefulness, rather than enhance performance. The current study also included a 5-min break prior to the last 3 trials of training, and in line with this stabilization account, we observed no change in performance for the RW condition after sleep. However, it remains unclear why we failed to observe performance decay in the TSD conditions. Importantly, while we failed to observe an effect of sleep on performance of these immediate trials, our data outline the importance of prior sleep to procedural performance across multiple subsequent practice opportunities throughout the following day.

Despite previous findings that napping can facilitate consolidation of previously learned motor sequences^9^,^10^,^12^,^22^,^23^, we failed to identify a similar effect when comparing nap and no-nap after TSD. This may relate to TSD prior to napping in our study, which is not typical of previous MSL studies investigating daytime naps^9^,^10^,^12^,^22^.

The literature exploring contributions from different sleep stages to procedural memory consolidation highlights a preferential role of NREM sleep^8^ and associated sleep spindles^12^,^24^,^25^, although some studies also indicate contributions from REM sleep^7^,^26^,^27^. In the present study, participants obtained almost exclusively NREM sleep (Table 1) during the afternoon nap and yet failed to demonstrate a sleep-dependent improvement in performance. Tentatively this may indicate a role for REM sleep^7^,^28^ or the occurrence of a full cycle between NREM and REM sleep stages^29^, both of which were disrupted in our experiment.

The absence of nap benefit could also have arisen from the fact that participants had performed multiple practices prior to the nap, resulting in atypical levels of pre-sleep performance. Procedural memory tasks are very sensitive to the amount of learning that occurs prior to sleep in order to show the relatively subtle improvements offered by a period of offline consolidation^30^. Our participants received three times the typical amount of finger tapping practice prior to napping^9^,^10^,^12^,^22^. However, this explanation is not tenable since performance in the two TSD conditions continued to lag behind that of the RW condition prior to the nap, which suggests gains were still achievable for the TSD conditions across the nap period.

Sleep inertia^31^ in the nap condition could also, in theory, mask subsequent finger tapping improvements. The majority of our participants awoke from SWS, which has been documented to cause the highest levels of sleep inertia^32^. However, the fact that Practice 3 took place 1.5 hours after the nap, and objective alertness was comparable between nap and no-nap only 30 minutes after waking from the nap (Supplementary Table 3), strongly argues against sleep inertia being contributory to a lack of improvement in the nap condition.

To our knowledge ours is the first study to explore nap effects on procedural memory after TSD, and we are aware of only one study that explored these benefits after partial sleep deprivation of 5-hours nocturnal sleep^33^. The majority have studied naps in well rested subjects^10^. However, it is important to note that naps are often taken to mitigate partial sleep deprivation accrued across one or multiple nights. Future work should identify whether naps are beneficial to procedural memory in the setting of partial sleep deprivation.

Interestingly, performance across all conditions significantly improved from the last practice episode to the one-week test. This is remarkable when you consider that there was no significant improvement across practices 1–4 for the RW condition, with near zero slope within each practice (Supplementary Fig. 1); yet a week with no practice was followed by significant gains. It thus appears that memory consolidation over a week with no additional practice provides benefits that are not evident during a day of multiple practice opportunities.

A week of offline consolidation after TSD thus appears to have alleviated its detrimental effects on performance. Prior studies exploring the long-term impact of post-training TSD on procedural memory have produced mixed results. One study found that TSD led to persistent deficits in performance of a texture discrimination task even after two nights of recovery sleep, while performance of well rested subjects continued to improve offline for up to 7 days^19^. Schönauer and colleagues^17^ recently demonstrated that performance deficit in a mirror tracing task after a night of TSD remained when tested one week later. However, the same study showed that finger tapping performance decrements typically associated with TSD^7^,^16^ disappeared after 3 or 6 nights of recovery sleep. Similarly, we showed that a week of offline consolidation eliminates the performance impairment associated with sleep deprivation.

Schönauer and colleagues suggested that the hippocampus may buffer information for subsequent consolidation at a time when sleep can be achieved, perhaps via physiological features of NREM sleep^2^ such as spindles^12^,^24^,^25^,^26^. This may explain the delayed gains we observed for TSD conditions. In our study, it was not possible to determine whether recovery sleep or simply the passage of time was responsible for offline improvement. However, given the clear deficits associated with TSD in next day performance, we could speculate that the occurrence of sleep at some point during the offline period was necessary for any improvement to occur.

The relative lack of improvement across multiple practice opportunities may suggest a ceiling effect, indicating that our task was not appropriate for exploring extended learning. However, against this, it is known that procedural learning elicits slow incremental gains in performance after initial skill acquisition^9^, and the observed significant RW and TSD improvements one week later show that slow gains were still possible. Moreover, as shown in Fig. 2A there was some recovery of TSD performance in the latter part of the day. This might reflect circadian or practice effects or a combination. The PVT is sensitive to such circadian effects, but only two were performed across the day, and it was not appropriate to compare them on account of their different durations. It was thus not possible to clarify whether this improvement reflects circadian or practice effects.

This study has some limitations. The fact that the RW training session was always the first session, and took place at home rather than in the lab, may have impacted on the way the task was performed. However, while early differences in performance trajectories during training support this possibility (Fig. 1C), performance during the last 3 trials of training on which our analyses were based was highly similar across conditions suggesting that differences had been greatly attenuated by this point. This suggests that the comparisons of conditions at this point in time and beyond are reasonable.

Furthermore, if at all, our design would bias performance in favor of the TSD conditions. Specifically, the benefit of additional training for the TSD conditions might be expected to attenuate differences between the TSD and RW conditions. Hence, the significantly lower performance after both TSD conditions relative to the RW condition provides robust evidence for a negative impact of TSD on procedural performance.

In conclusion, our results suggest that a night of TSD has a sustained negative impact on procedural performance the following day that is not alleviated by a 60-minute afternoon nap or repeated practice. However, after a week without practice procedural performance deficits disappear.

## Methods

### Participants

Thirty-seven right-handed participants took part in the study. Participants were excluded if they had a history of neurological or sleep disorders, had extreme morningness-eveningness preferences with scores less than 30 or more than 70^34^, had a history of obstructive sleep apnea, consumed more than 2 caffeinated drinks a day, smoked, or habitually slept less than 6.5 hours a night. Twelve subjects failed to complete all three conditions. Data from seven participants were excluded as they either did not follow the instructions (1), received >10 years of intensive piano training (1), did not complete 1 or more practices (3), or did an interfering sequence prior to sleep in the RW session (2). The final sample consisted of 18 participants between the ages of 19 and 25 (mean age 21.78 ± 1. 8 years; 11 females). Previous work has shown that this sample size is sufficient to demonstrate the effects of TSD on MSL^7^. Participants provided written informed consent, in compliance with a protocol approved by the National University of Singapore Institutional Review Board and received monetary compensation after completion of all conditions. Methods were carried out in accordance with the approved protocol.

### Experimental procedure

In each experimental condition, participants were required to maintain a consistent sleep pattern (8 hours time-in-bed (TIB); going to bed before 01:00 and waking before 09:00) in the week prior to each condition, confirmed with sleep diaries and actigraphy in the three nights prior to each condition (actigraphy-estimated TIB = 8 hours 8 mins ± 8 mins, average sleep timing 00:11–08:20). Participants were reminded to abstain from napping, consuming any medications, alcohol or caffeine 24 hours prior to and during each condition. Each condition began with training that started at 22:00.

Training took place in the participant’s home for the RW condition, and in the lab for both TSD conditions. In the RW condition training was followed by an 8 hour TIB sleep opportunity at home, assessed with actigraphy. For TSD conditions, participants remained in the lab where they were monitored overnight to ensure they did not sleep. During the night they were permitted to read and watch movies, and were asked to perform a test battery at 2-hour intervals (23:00, 01:00, 03:00, 05:00, 07:00 and 09:00), data of which were not analysed in this study. The test battery lasted approximately 25 mins, and included 7 tasks presented in the following order: Karolinska Sleepiness Scale (KSS), Sustained Attention to Response Task (SART), Symbol Digit Modalities Test (SDMT), verbal 1 and 3-back tasks, Mental Arithmetic Test (MAT), Positive and Negative Affect Scale (PANAS), and a 10-minute Psychomotor Vigilance Task (PVT).

In all three conditions, participants performed 4 practices of the SFTT across the following daytime period. During the training phase, participants were not blind to the RW/TSD conditions, but for Practices 1–2 following TSD nights, they were blind to the nap/no-nap conditions. Experimenters were not blind to the conditions. All conditions involved performing a test battery at 11:00. Participants were monitored in the laboratory by experimenters to restrict surreptitious napping. During the day participants also performed additional tests of declarative memory that are not reported here, including a spatial memory task and word-pair associates task. The schedule for performing these tasks was identical across conditions. Participants returned to perform a 3-trial re-test of the SFTT one-week later.

### Sequential finger tapping task (SFTT)

Participants learned an eight-element sequence (Fig. 1B) by pressing four numeric keys, using all four fingers of their non-dominant hand as quickly and accurately as possible. The numeric sequence was displayed on screen, and position within the sequence was shown by hashtags appearing underneath the sequence. Experimental stimuli were presented using E-Prime 2.0 (Psychology Software Tools, Pittsburgh, PA) on Acer Aspire E11 computers.

Within each experimental condition, participants engaged in one training period in the evening and four practices the following day (Fig. 1A), each lasting 19.5 minutes. Each included 12 trials of 30-sec sequence performance interspersed with 30-sec of rest. This was followed by a 5-minute break before 3 further trials were performed – 15 trials in total^13^,^14^,^15^. Each condition concluded with performance on three trials of a novel sequence, to assess for non-sequence specific gains in motor speed. Participants returned one week later and were tested on 3 trials of the trained sequence. For each participant, three pairs of eight-digit sequences (one trained and one novel) were randomly assigned to the three conditions. Sequences were of comparable difficulty but dissimilar from one another to minimise transfer of learning between sequences (Supplementary Table 2). Each sequence began and ended on the same digit, included no repeated items within the sequence (e.g., 1–1), and each digit appeared twice (Supplementary Table 1).

### Psychomotor Vigilance Task

Participants performed a psychomotor vigilance task (PVT) at various time points during the experiment to measure levels of sustained attention. Participants were required to respond as quickly as possible with a key press whenever they observed a counter to appear on screen. This appeared at random intervals ranging between 2000 msec and 10,000 msec, and was followed by a beep if no response was made within 10,000 msec. The reciprocal RT (1/RT) was used as a measure of sustained attention. For TSD conditions, 10-min PVT’s were performed at 23:00, 01:00, 03:00, 05:00, 07:00 and 09:00, data not presented. In all conditions a 10-min PVT was performed at 11:00, and a 3-min PVT at 16:30. There was one missing data point for the PVT at 11:00 due to error in data recording.

### Polysomnography

Polysomnography (PSG) was recorded using a SOMNOtouch recorder (SOMNOmedics GmbH, Randersacker, Germany) with six EEG channels, C3, C4, M1, M2, Cz (reference) and FPz (ground). C3 and C4 were then referenced to contralateral mastoids for scoring purposes. We also recorded electrooculography (EOG) and submental electromyography (EMG). Impedance was kept below 10 kΩ for all electrodes. Signals were sampled at 256 Hz. Scoring was performed visually by trained technicians using the FASST toolbox (http://www.montefiore.ulg.ac.be/~phillips/FASST.html) in accordance to rules set by the American Association of Sleep Medicine (AASM)^35^.

### Actigraphy

To ensure that sleep history was comparable across conditions, participants’ sleep one week prior to each condition was recorded using daily sleep logs and wrist actigraphy (Actiwatch 2, Respironics, Inc.), Analysis focused on 3-nights prior to each condition. Data were collected at 2 min resolution and were scored with Actiware software (version 6.0.2, Respironics, Inc.). TIB was calculated using a medium sensitivity threshold.

### Statistical analyses

Statistical analyses were performed using SPSS version 22. Speed was defined as the number of correct sequences completed within a 30-sec trial, and error was the number of incorrect responses within a 30-sec trial. These were averaged across trials 1–15 in each round of training and practice. Mean percentage of improvement during practices was calculated relative to the mean pre-retention training performance. We ran a two-way repeated measures ANOVA with practice (P1-P4) and condition (RW/TSD nap/TSD no-nap) as within-subjects factors. Greenhouse-Geisser corrections were reported whenever the assumption of sphericity was violated. Planned post-hoc comparisons were performed between RW and combined TSD conditions, as well as between TSD nap and TSD no-nap conditions. To assess the influence of napping in the TSD conditions, the combined mean of pre-nap practices (P1 and P2) were compared to the mean of combined post-nap practices (P3 and P4). Statistical effects reported here have not been replicated in a different sample. All reported p-values reflect the results of two-tailed t-tests.

PVT performance was assessed by calculating the reciprocal RT (1/RT). The effect of condition on it was correlated with the effect of condition on SFTT using Pearson’s correlation.

### Code availability

Codes used to convert .txt output files into .mat files and those to calculate mean performance per condition are available upon request.

## Additional Information

**How to cite this article**: Kurniawan, I. T. *et al*. Procedural performance following sleep deprivation remains impaired despite extended practice and an afternoon nap. *Sci. Rep.* **6**, 36001; doi: 10.1038/srep36001 (2016).

**Publisher’s note:** Springer Nature remains neutral with regard to jurisdictional claims in published maps and institutional affiliations.

## Supplementary Material

Supplementary Information

## Figures and Tables

**Table 1:** Mean (±SEM) of sleep data during 1-hour nap opportunity in minutes (*n* = 18).

Sleep Variables	Mean (±SEM)
Time in bed (TIB)	60.05 ± 0.17
Total sleep time (TST)	54.05 ± 1.19
Sleep onset	6.88 ± 1.15
Stage 1	0.13 ± 0.08
Stage 2	9.63 ± 1.68
SWS	43.16 ± 2.69
Total NREM	52.94 ± 1.32
REM	1.11 ± 0.50
WASO	0.11 ± 0.05
Sleep Efficiency (%)	90.18 ± 1.94
