# Supplementary material for: Procedural performance following sleep deprivation remains impaired despite extended practice and an afternoon nap
Source: Sci Rep. 2016 Oct 26;6:36001. doi: 10.1038/srep36001 (PMC5080542; doi:10.1038/srep36001)
Supplement: Supplementary Information [file srep36001-s1.doc]

**Supplementary Materials**

**Procedural performance following sleep deprivation remains impaired despite extended practice and an afternoon nap**

Irma Triasih Kurniawan ^1*^, James Nicholas Cousins ^1*^, Pearlynne L.H. Chong ^1^ &, Michael W.L. Chee ^1^


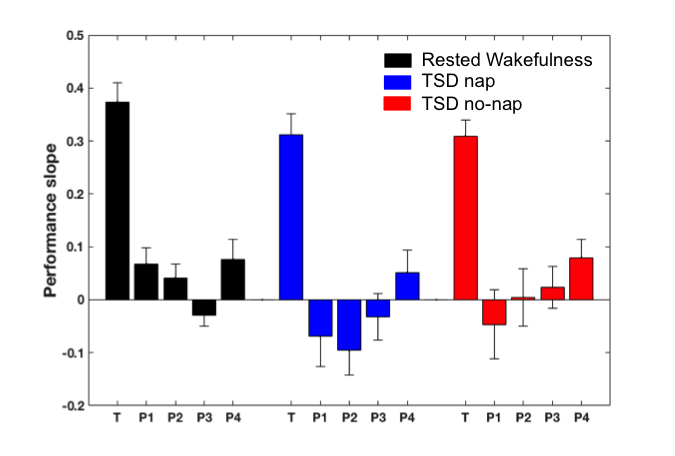


**Supplementary Figure 1.** Finger tapping performance as measured by the slope of improvement in performance speed across 15 trials for training (T) and Practice (1-4) for each condition. These plots show increasing performance across 15 trials during training but relatively flat performance improvement in practice rounds. Mean + SEM. We first ran subject-wise linear regression with trial number (1-15) predicting speed (# correct seq/ 30 s) for each Training or Practice round. The regression weights were entered into a 3 Condition (RW/TSD nap/TSD no-nap) x 5 Round (T/P1-4) repeated measures ANOVA, yielding a non-significant main effect of condition (*F*(2,34)=3.2, *p=*0.053) and condition-by-round interaction (*F*(8,136)=1.02, *p=*0.42), but a significant main effect of round (*F*(4,68)=42.7, *p<*0.0001) which was driven by a significantly higher slope during Training compared to the average of Practice 1-4 (*t*(17)=15.5, *p<*0.0001). Rerunning the ANOVA without the Training round also yielded non-significant effect of condition or interaction (*F*(2,34)=2.16, *p=*0.13; *F*(6,102)=1.3, *p=*0.26) and a significant effect of practice round (*F*(3,51)=3.04, *p=*0.037). This analysis suggests that performance within each practice in TSD did not decline more rapidly than in RW.


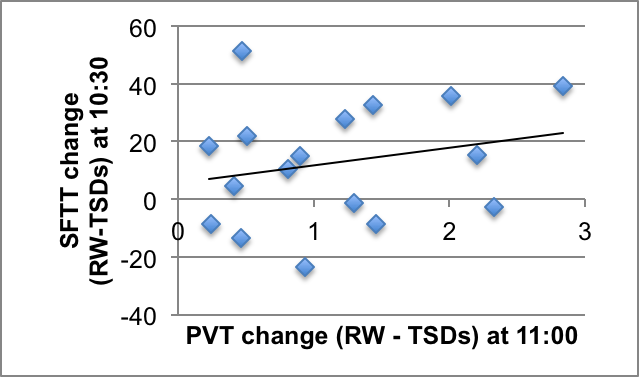


**Supplementary Figure 2.** The effect of condition (RW>TSDs) on SFTT in Practice 1 (10:30) was not significantly correlated with its effect on a 10-min Psychomotor Vigilance Task (11:00) (*r*(16)=0.23, *p*=0.3).


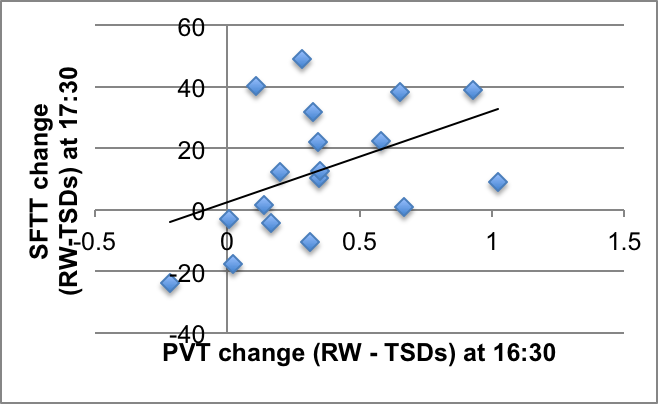


**Supplementary Figure 3.** The effect of condition (RW>TSDs) on SFTT in Practice 3 (17:30) was not significantly correlated with its effect on a 3-min Psychomotor Vigilance Task (16:30) (*r*(17)=0.4, *p*=0.06).

**Supplementary Table 1.** Original and novel sequential finger tapping sequences, randomized across three experimental conditions for each participant.

| Set | Name | Eight-digit sequence | | | | | | | |
| --- | --- | --- | --- | --- | --- | --- | --- | --- | --- |
| 1 | Seq 1 | 1 | 4 | 3 | 2 | 3 | 4 | 2 | 1 |
|  | Novel 1 | 3 | 2 | 4 | 1 | 4 | 2 | 1 | 3 |
| 2 | Seq 2 | 4 | 1 | 3 | 2 | 3 | 1 | 2 | 4 |
|  | Novel 2 | 1 | 2 | 4 | 3 | 4 | 2 | 3 | 1 |
| 3 | Seq 3 | 2 | 3 | 4 | 1 | 4 | 3 | 1 | 2 |
|  | Novel 3 | 3 | 1 | 2 | 4 | 2 | 1 | 4 | 3 |

**Supplementary Table 2.** Means (+ SEM) of slope of improvement in finger tapping speed across trials 1-15 during training for each sequence in the RW session. A one-way between subjects ANOVA showed comparable learning rates across groups of participants who performed sequence 1, 2 or 3 in the RW session.

| Sequence 1 | Sequence 2 | Sequence 3 | F test |
| --- | --- | --- | --- |
| 0.36 + 0.04 | 0.38 + 0.06 | 0.38 + 0.09 | *F*(2,17)=0.03, *p*=0.9 |

**Supplementary Table 3.**  Reciprocal RT (1/RT) performance in a 10-min and 3-min Psychomotor Vigilance Task. Mean (SEM).

| **Effects** | **n** | **RW** | **TSD nap** | **TSD no-nap** |
| --- | --- | --- | --- | --- |
| 10-min (at 11:00) | 17 | 3.61 (0.07) | 2.42 (0.19) | 2.48 (0.19) |
| 3-min (at 16:30) | 18 | 3.69 (0.08) | 3.41 (0.1) | 3.28 (0.12) |
